# Bosutinib inhibits migration and invasion via ack1 in kras mutant non-small cell lung cancer

**DOI:** 10.1186/1476-4598-13-13

**Published:** 2014-01-24

**Authors:** Daniel SW Tan, Benjamin Haaland, Jia Min Gan, Su Chin Tham, Indrajit Sinha, Eng Huat Tan, Kiat Hon Lim, Angela Takano, Sai Sakktee Krisna, Minn Minn Myint Thu, Hoe Peng Liew, Axel Ullrich, Wan-Teck Lim, Boon Tin Chua

**Affiliations:** 1 Department of Medical Oncology, National Cancer Centre Singapore, Singapore, Singapore; 2 Cancer Therapeutics Research Laboratory, National Cancer Centre Singapore, Singapore, Singapore; 3 Centre for Quantitative Medicine, Office of Clinical Sciences, Duke-NUS Graduate Medical School, Singapore, Singapore; 4 Department of Statistics and Applied Probability, National University of Singapore, Singapore, Singapore; 5 Singapore OncoGenome Laboratory, Institute of Medical Biology, Agency of Science Technology and Research, Singapore, Singapore; 6 Biomedcore Inc., 1580 Rossi Drive, Tecumseh ON N9A 6 J3, Canada; 7 Department of Pathology, Singapore General Hospital, Singapore, Singapore; 8 p53 Laboratory, Biomedical Science Institutes Agency of Science Technology and Research, Singapore, Singapore; 9 Max-Planck Institute of Biochemistry, Am Klopferspitz, Martinsried, Germany; 10 INM-SOG, Institute of Molecular and Cell Biology, Agency of Science Technology and Research, Singapore, Singapore

**Keywords:** Lung cancer, ACK1, KRAS, Bosutinib, Metastasis

## Abstract

The advent of effective targeted therapeutics has led to increasing emphasis on precise biomarkers for accurate patient stratification. Here, we describe the role of ACK1, a non-receptor tyrosine kinase in abrogating migration and invasion in KRAS mutant lung adenocarcinoma. Bosutinib, which inhibits ACK1 at 2.7 nM IC_50_, was found to inhibit cell migration and invasion but not viability in a panel of non-small cell lung cancer (NSCLC) cell lines. Knockdown of ACK1 abrogated bosutinib-induced inhibition of cell migration and invasion specifically in KRAS mutant cells. This finding was further confirmed in an *in vivo* zebrafish metastatic model. Tissue microarray data on 210 Singaporean lung adenocarcinomas indicate that cytoplasmic ACK1 was significantly over-expressed relative to paired adjacent non-tumor tissue. Interestingly, ACK1 expression in “normal” tissue adjacent to tumour, but not tumour, was independently associated with poor overall and relapse-free survival. In conclusion, inhibition of ACK1 with bosutinib attenuates migration and invasion in the context of KRAS mutant NSCLC and may fulfil a therapeutic niche through combinatorial treatment approaches.

## Introduction

Lung cancer is the leading cause of cancer related death world-wide [1]. It is estimated there will be ~228 190 new lung cancer cases and ~159 480 lung cancer deaths in the United States in 2012 (http://seer.cancer.gov/statfacts/html/lungb.html). Although an increasing number of genetic alterations in non-small cell lung cancer (NSCLC) have become amenable to targeted therapeutics [2], an emerging challenge is the identification of co-factors that can modulate and attenuate intracellular signaling cascades implicated in drug response or resistance–so as to enable better patient selection and rational drug combinations [3].

Activated Cdc42 associated kinase (ACK1) is a non-receptor tyrosine kinase residing in the cytoplasm. The kinase domain is located at the N-terminal half of the protein followed by multiple protein-protein interaction domains such as CRIB where Cdc42 binds [4]. The C-terminal half of the protein interacts with partners including clathrin, EGFR, ubiquitin, Nedd4 E3 ligase and Grb2 [5-8]. Recent work by Mahajan *et al.* shows that ACK1 phosphorylates AKT at Tyr 176, resulting in its activation [9]. Several reports have implicated ACK1 over-expression and amplification in tumorigenesis of different tissue types e.g. gastric, pancreatic and lung [10,11]. High expression of phosphorylated ACK1 correlates with disease progression in breast, prostate and pancreatic cancers [12-14], with specific interactions between the ACK1 kinase and key signaling nodes e.g. androgen receptors in prostate cancer. In melanoma cell lines, ACK1 is activated in response to integrin signaling, resulting in cell spreading [15]. *In vivo*, ACK1 over-expressing cells resulted in aggressive metastatic tumors [10,12] while *in vitro* silencing of the *ACK1* gene in RAS-transformed NIH3T3 cells increased apoptosis [16]. Recently, we have also shown that silencing of ACK1 results in reduced ERK and AKT phosphorylation and interestingly, EMT reversion [17].

We hypothesized that ACK1 hyperactivity through over-expression influences metastatic potential in lung adenocarcinoma and can be targeted with kinase inhibitors. Bosutinib (SKI-606) is a third generation dual SRC-ABL kinase inhibitor developed by Wyeth (Pfizer) that also binds and prevents auto-phosphorylation of ACK1 at IC50 of 2.7 nM [18,19]. Our results show that bosutinib inhibited cancer cell migration and invasion via ACK-1 in a KRAS dependent manner – in both *in vitro* cell lines as well as an *in vivo* zebrafish model. Further, we validated ACK1 protein expression in 210 lung adenocarcinoma tissue microarrays using immunohistochemistry, where high expression of tumor ACK1 was observed as compared to paired adjacent “normal” lung tissue. Although tumor ACK1 expression was not associated with survival outcomes in resected NSCLC, intriguingly, ACK1 expression in adjacent “normal” lung was associated with worse overall and relapse-free survival in both univariate and multivariate models.

## Results

### Bosutinib inhibits KRAS mutant but not KRAS wild type cell migration and invasion

**Figure 1:**
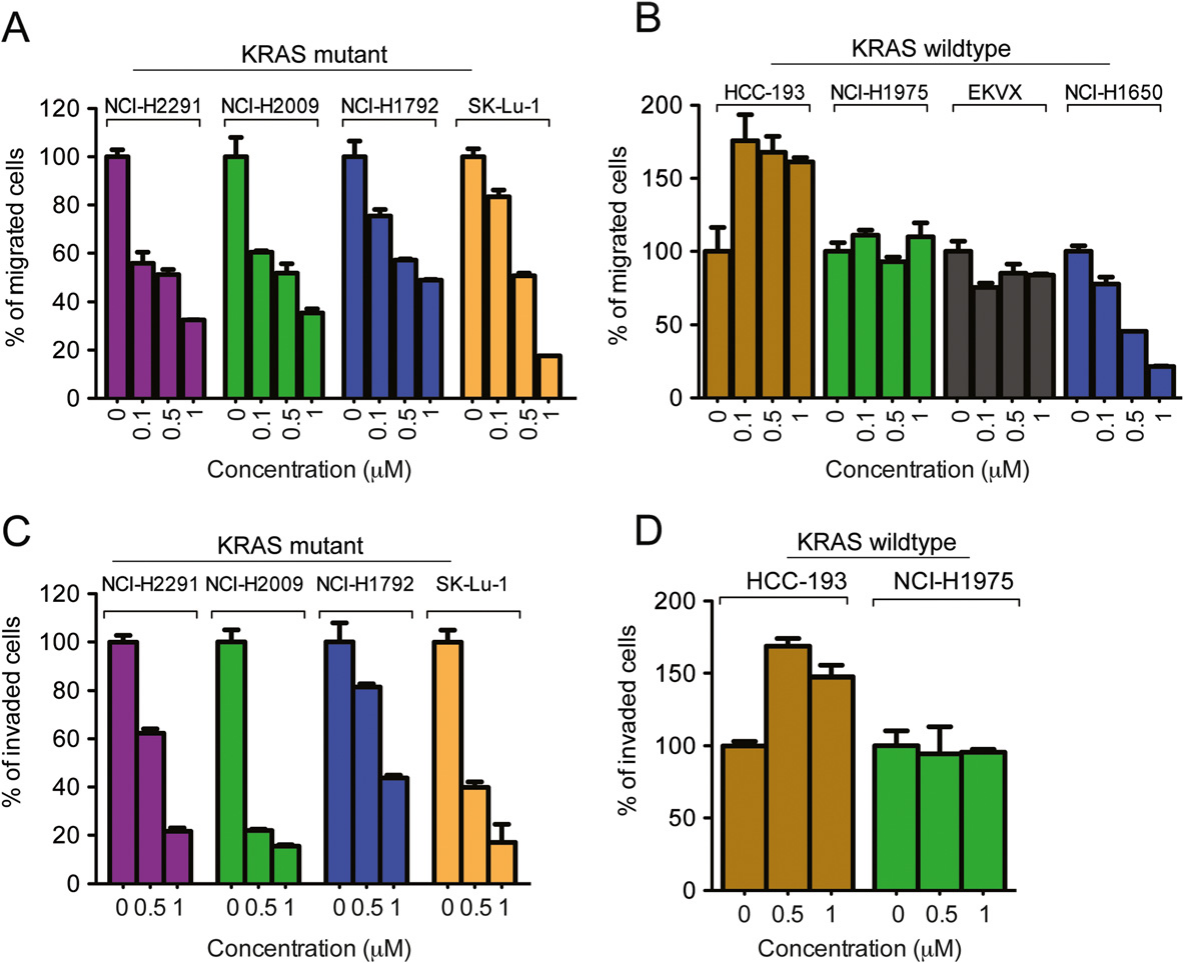
***Bosutinib inhibit KRAS mutant cell migration and invasion but not KRAS wildtype cells.*** The serum-starved KRAS **(A & C)** mutant and **(B & D)** wildtype cells were trypsinized and seeded in the upper chamber of the Transwell (8 mm pore, **A** &**B**) or Matrigel™ **(C & D)**, in the presence of DMSO or bosutinib at various concentrations (0.1, 0.5 and 1 μM). Medium containing 10% FBS and DMSO or bosutinib (0.1, 0.5 and 1 μM) was used as chemoattractant in the lower chamber. The migrated or invaded cells were fixed and stained with 0.5% crystal violet blue after 6 h and 24 h respectively. Cells that migrated or invaded across the filter were counted. Experiments were carried out in duplicates with five random fields counted. The percentage of migrated or invaded cells was expressed with respect to the DMSO treated cells.

We have previously demonstrated that ACK1 plays an important role in cell migration and epithelial mesenychmal transition in both *in vitro* over-expression and gene silencing systems [17]. We tested the effect of bosutinib on cell migration in a panel of eight NSCLC cell lines that migrate efficiently across the 8 μm transwell with 10% FBS as a chemoattractant. We also tested the invasive potential of the cell lines using Matrigel™ assay. As shown in Figure 1, sub-lethal concentration (0.1, 0.5 and 1 μM) of bosutinib were sufficient to inhibit cell migration and invasion in a dose-dependent manner. Unexpectedly, this was only observed in KRAS mutant cells as shown in Figure 1A and C. In contrast, bosutinib had no effect on migration in 3 out of 4 KRAS wild type NSCLC cell lines (Figure 1B). In addition, all 4 KRAS mutant cell lines showed reduced migration in the invasion Matrigel assay, while two KRAS WT cell lines tested were not inhibited by bosutinib (Figure 1D).

### Effect of bosutinib on viability of NSCLC cell lines is independent of KRAS status

**Figure 2:**
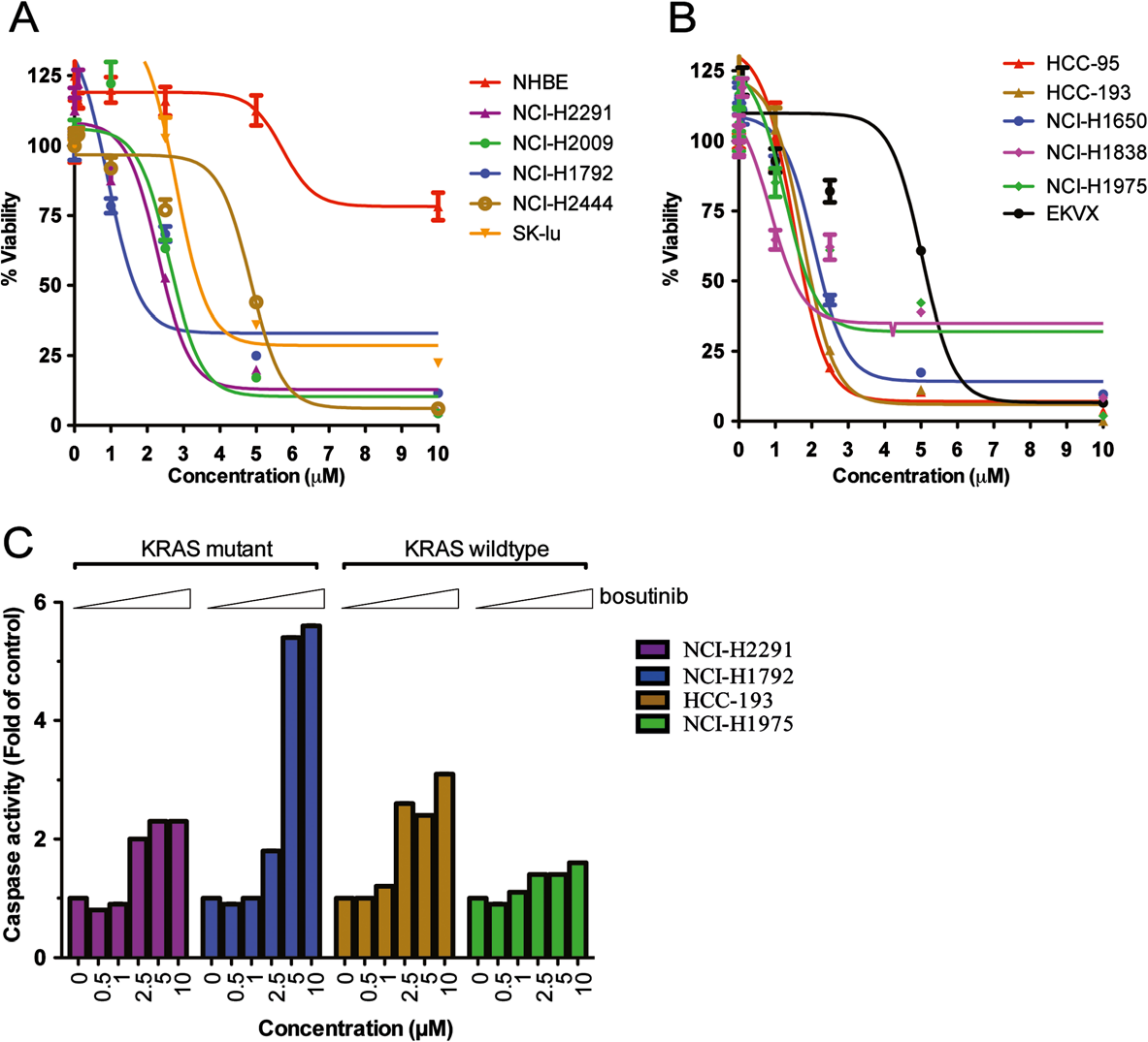
***Effect of bosutinib on viability of NSCLC cell lines is independent of KRAS status.*** The KRAS mutant **(A)** and wildtype **(B)** NSCLC or Normal Human Bronchial Epithelial (NHBE) cells were seeded onto 96-well plates for 24 h. The cells were treated with various concentrations (0–10 μM) of bosutinib for 72 h followed by cell viability assay measured by CellTitre Glo. The cell viability results were normalized to DMSO-treated controls and expressed in percentage. **(C)** Four NSCLC cells were treated with bosutinib for 72 h at different concentrations. Caspase3/7 activities were measured using Caspase-Glo 3/7 luminescent assay kit. The enzymatic activity is expressed as the value with respect to untreated cells.

Across the panel of NSCLC lines, bosutinib reduced cell viability at micromolar IC_50_ of between 1–5 μM (Figure 2A and B) *via* apoptosis (Figure 2C) in all tested KRAS mutant and KRAS wild type (WT) lung cancer cell lines. Based on steady state (D15) dosing of bosutinib [MW 530.44616] in the clinic at 400 and 500 mg daily, Cmax was 190 ng/mL and 273 ng/mL respectively, [20] approximately corresponding to *in vitro* concentrations of 0.3 – 0.5 μM. Thus, clinically relevant doses of bosutinib are able to inhibit migration and invasion but not viability in NSCLC cell lines.

### Bosutinib inhibition of cell migration and invasion is ACK1 dependent and SRC-independent

**Figure 3:**
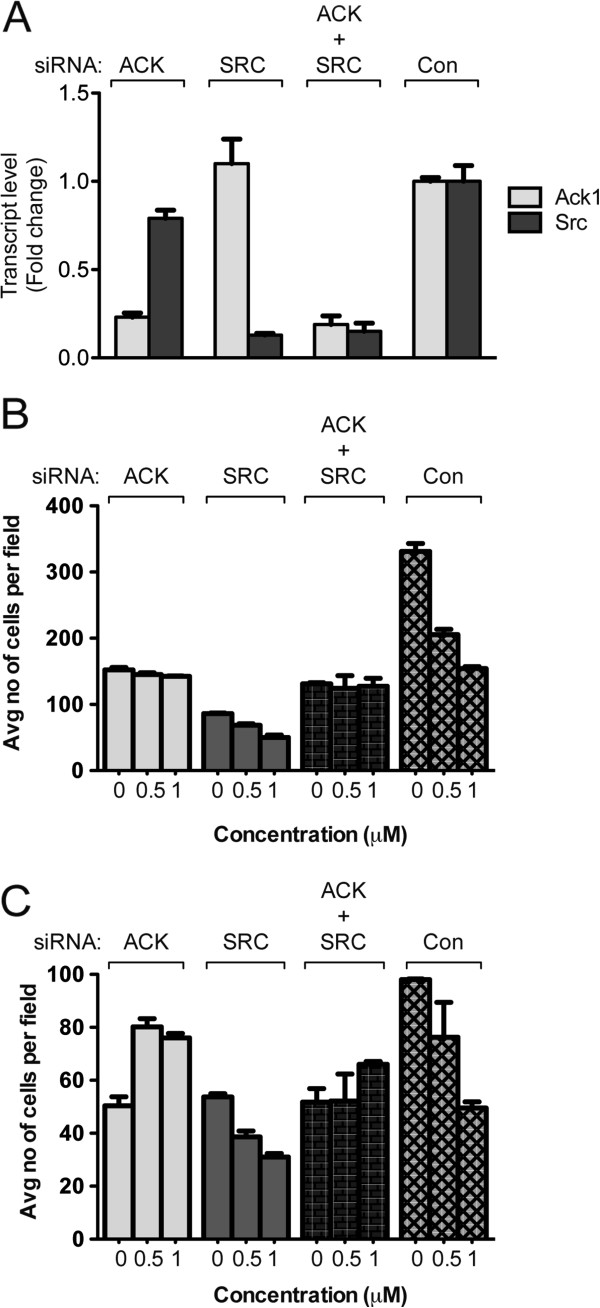
***Bosutinib inhibit cell migration and invasion is ACK1 dependent and SRC- independent.*** NCI-H1792 were transfected with siRNA (control, ACK1 or SRC) using Oligofectamine for 72 h. The cells were harvested at 72 h. **(A)** Quantitative real-time PCR analysis was performed using 20 ng of cDNA and respective primers set for *ack1* and *src* and normalized to *gapdh*. The serum-starved cells were trypsinized and seeded in upper chamber of the Transwell (8 mm pore, **B**) or Matrigel™ **(C)**, in the presence of DMSO or bosutinib at various concentrations (0.5 and 1 μM). Medium containing 10% FBS and DMSO or bosutinib (0.5 and 1 μM) was used as chemoattractant in the lower chamber. The cells were fixed and stained with 0.5% crystal violet blue after 6 h (migration, **B**) and 24 h (invasion, **C**). Cells that migrated or invaded across the filter were counted. Experiments were carried out in duplicates with five random fields counted.

Since both SRC and ACK1 play a role in cell motility [17,21-24] and bosutinib can inhibit both kinases, we evaluated if one or both of these target kinases (ACK1 and SRC) were mediating the migratory effect. Using NCI-H1792, a KRAS mutated NSCLC cell, we silenced either ACK1 or SRC or both and measured migration and invasion upon bosutinib treatment. Real time PCR data (Figure 3A) showed that silencing of ACK1, SRC or both effectively reduced the respective transcript levels. Expectedly, with ACK1 or SRC knockdown, cell migration and invasion was reduced ~50%, further consolidating the role of these kinases in cell motility (Figure 3B & C). In SRC-silenced cells, dose-dependent inhibition of cell migration and invasion was still observed after treatment with increasing concentration of bosutinib. In contrast, in ACK1 and ACK1/SRC dual-silenced cells, bosutinib-induced inhibition of cell migration was abolished. The same effect was also observed in a second KRAS mutant NSCLC cell line, SK-Lu-1 (Additional file 1: Figure S1) after treated with bosutinib. These data support the role of ACK1 as a target of bosutinib in inhibiting cell migration and invasion in KRAS mutant cell lines.

### Reduction of lung cancer cell metastasis in zebrafish embryos by bosutinib is ACK1 dependent

**Figure 4:**
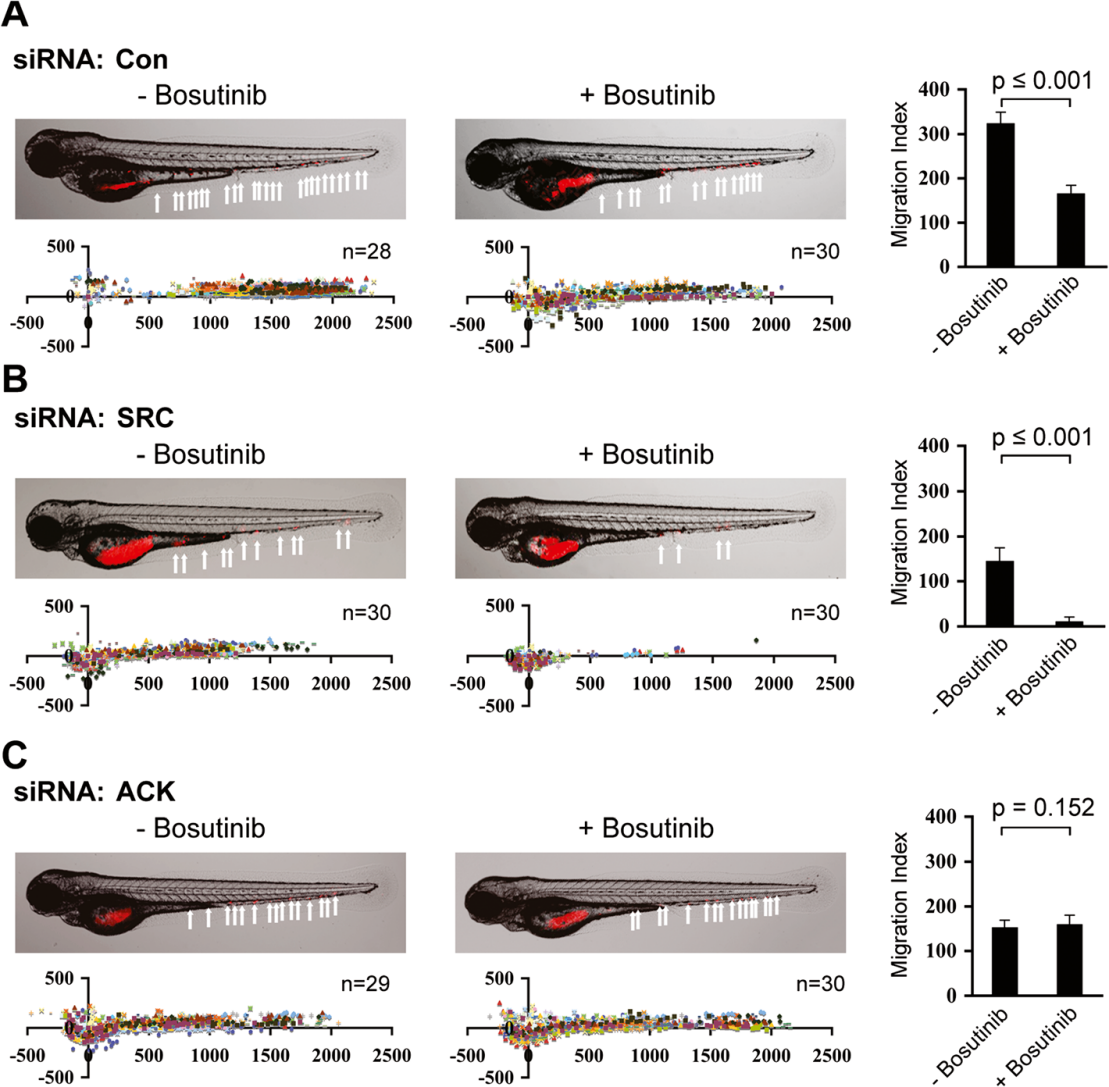
***Reduction of lung cancer cell metastasis in zebrafish embryos by bosutinib is ACK1 dependent.*** Embryo injected with Vibrant DiD labeled NCI-H2009 cells showing tumor foci burden determined by segmented red channel (upper) and scatter plot representation of cell dissemination (bottom). Each data series represents one fish and number of injected embryos from 3 biological replicates is indicated (n). Comparative inhibition of cell migration, as measured by Migration Index, between untreated and drug pretreated cells is represented as bar graph (right), Mean ± SD. **(A)** Embryos injected with NCI-H2009 transfected with control siRNA. **(B)** Embryos injected with NCI-H2009 transfected with SRC siRNA. **(C)** Embryos injected with NCI-H2009 transfected with ACK1 siRNA.

Next, we examined if *in vitro* migratory and invasive inhibition by bosutinib could be translated into metastasis inhibition *in vivo*. Zebrafish is a well-recognized model of human malignancy due to conserved genetics and cell biology [25]. Using zebrafish as the host organism, we have developed a quantitative *in vivo* cell migration assay to monitor the response of cells to anti-cancer agents. Transparency of the embryos provides us with the unique ability to visualize *in vivo* drug responses and migratory changes of cancer cells in real time. Here we injected boluses of fluorescently labeled human NCI-H2009 cells, which were successfully transfected with siRNA against ACK1, SRC or negative control (Additional file 2: Figure S2), into the yolk sac of 24–30 h old embryos. The labeled NCI-H2009 cells were observed to migrate out from the injection site and metastasized to various parts of the embryos (Figure 4A). To test the effect of bosutinib on these metastatic cells *in vivo*, we pretreated the labeled cells with bosutinib before injecting them into the embryos. The metastatic potential of NCI-H2009 cells transfected with control and SRC siRNA was significantly reduced after pretreatment with low dose bosutinib (Figure 4A and B) while such a significant reduction was not observed in cells transfected with ACK1 siRNA (Figure 4C). These observations further support the role of ACK1 as a target of bosutinib in inhibiting cell migration and invasion in KRAS mutant cell lines *in vivo*.

### ACK1 is highly expressed in lung adenocarcinoma

**Figure 5:**
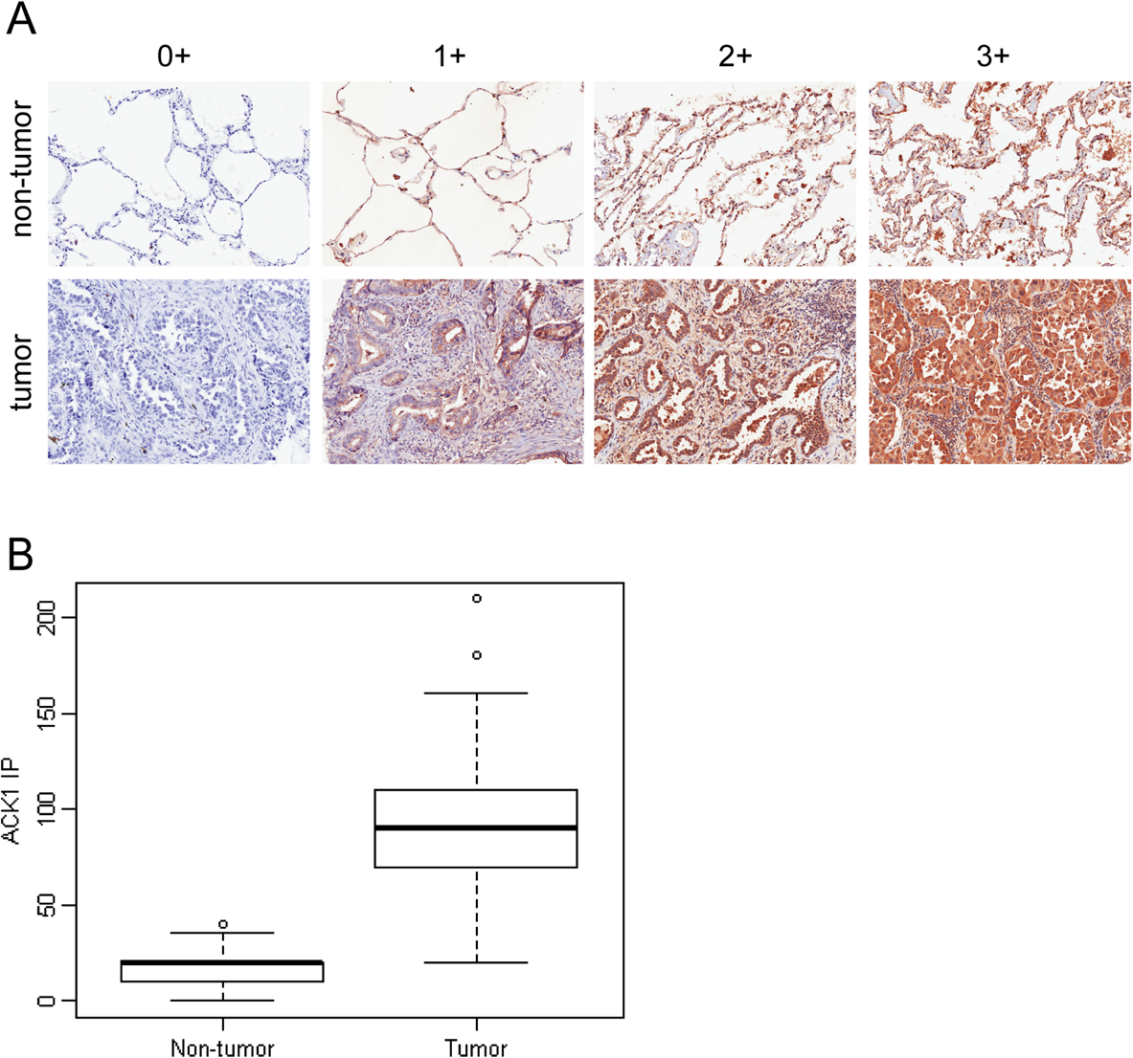
***ACK1 is highly expressed in lung adenocarcinoma.*** Immunohistochemistry staining was performed on 210 NSCLC tumor and paired non-tumor sections on an in-house TMA using anti-ACK1 (C20, see material and method). **(A)** ACK1 immuno-positivity was defined as presence of brown cytoplasmic staining. Staining intensity was scored as 0, 1+, 2+ and 3+ (no, weak, moderate and strong staining, respectively). **(B)** Percentage of positively stained tumor cells was assessed as proportion of total number of tumor cells present in the section. Intensity percentage score, IP, was defined as product of the maximum immunostaining intensity and percentage of tumor cells stained.

In 2005, Van der Horst *et al.*[10] showed that the ACK1 gene is amplified in numerous cancer types including lung cancers and this correlated with metastatic potential and poor prognosis. Here, we validated the total protein expression of ACK1 using immunohistochemistry staining on 210 lung adenocarcinoma on an in-house tissue microarray (TMA). In comparison with surrounding paired non-tumor lung sections, ACK1 is substantially and significantly (all p < 0.001) over-expressed in tumor tissue across immunohistochemisty summaries (including ACK1 maximum staining intensity, percentage staining, and IP). The difference in IP score (see Immunohistochemistry section in Method for definition) between tumour and adjacent non-tumour lung tissue is illustrated in Figure 5.

### ACK1 over-expression in adjacent “normal” lung tissue portends poorer outcomes in lung adenocarcinoma

The ACK1 IHC data was subjected to statistical analysis. Here, we found significant univariates that predicted for overall and relapse-free survival were stage, grade, smoking status, histology, and non-tumor ACK1 expression, as shown in supplementary data, Additional file 3: Table S1. Multivariate models were developed, adjusting for the relationship between prognosis and the important clinical covariates stage and grade, in the context of a Cox proportional hazards model. Interestingly, ACK1 over-expression in paired surrounding non-tumor “normal” tissue was independently associated with worse overall and relapse-free survival, as shown in Table 1.

**Table 1:** Unique relationship^*^between overall and relapse-free survival and the ACK1 IP in tumor and non-tumor tissues

	**Overall survival**	**Relapse-free survival**
**Tumor ACK 1 IP**	p = 0.982 (overall) (per 10 units) 0.999 (0.930-1.074)	p = 0.719 (overall) (per 10 units) 0.987 (0.916-1.062)
**Non-tumor ACK 1 IP**	p < 0.001 (overall) (per 10 units) 2.002 (1.370-2.927)	p < 0.001 (overall) (per 10 units) 1.907 (1.328-2.737)

^*^Adjusted for stage and grade.

## Discussion

In the era of molecularly-directed therapeutics in NSCLC, there has been increasing interest in discovery of novel biomarkers for improved patient stratification and treatment. While EGFR mutant and ALK translocated NSCLC have been successfully targeted in the clinic, KRAS mutations account for 15-22% of cases and to date, there are no clinically validated targeted therapies [2,26]. In this article, we present novel data highlighting a potential therapeutic role for bosutinib in abrogating migration and invasion in KRAS mutant NSCLC cell lines through ACK1.

The unexpected observation of bosutinib-mediated attenuation of migration and invasion specifically in KRAS mutant cell lines underscores the importance of evaluating targeted therapeutics in the appropriate genetic context. Our experiments also reveal the potential for identifying therapeutic niches beyond oncogenic drivers, where “off-target” inhibition of selected kinases may confer desirable anti-cancer effects other than reducing cell viability or induction of apoptosis. Indeed, the induction of apoptosis at bosutinib doses beyond 1 μM very likely involves non-specific multi-kinase inhibition. In contrast, inhibition of migration and invasion was observed at doses as low as 0.1 μM, consistent with bosutinib-specific “on-target” kinase inhibition at clinically relevant doses (Cmax of bosutinib at 400 mg daily is 190 ng/mL which is approximately 0.3 μM).

In this study, silencing of ACK1 or SRC alone significantly reduced cell migration and invasion, confirming their role in regulating metastasis. In addition, using bosutinib at dose of 0.5 μM and 1 μM)) as a chemical probe, we observed that the effect on migration and invasion were abrogated only by knockdown of ACK1 and not SRC, highlighting the importance of ACK1 in the signaling axis. This is further supported by the zebrafish model, where siRNA to ACK1 abrogated the anti-migratory effect of bosutinib. Another previously reported interacting partner of ACK1, AXL has been shown to regulate cell migration and invasion in breast cancer. In our NSCLC panel, we found that absence of AXL in NCI-H1792 had no effect on bosutinib-induced inhibition in cell migration and invasion (Additional file 4: Figure S3), suggesting that contradistinct to breast cancer [27]. AXL may not play a dominant role in the metastatic phenotype in NSCLC. Furthermore, given that ACK1 can be activated by receptor tyrosine kinases such as EGFR and ALK [5,24], bosutinib may have broader therapeutic relevance to NSCLC.

Separately, through the assembly of 210 tumors, we created TMAs to examine the clinical relevance of ACK1 expression in NSCLC. Here we chose to focus on total ACK1 expression and not phospho-ACK1 for two reasons. First, we have shown previously that in cell lines over-expression of wild type ACK1 plasmid resulted in ACK1 that is phosphorylated and active [17]. Secondly, the commercial available phospho-ACK1 antibody is not optimized for our TMAs, and artefactual dephosphorylation due to inconsistencies in time to fixation of paraffin embedded surgical samples may limit the accuracy of depicting intrinsic signaling activity [28,29]. In our cohort of 210 patients with lung adenocarcinoma, there was a substantial and significant difference in ACK1 protein expression between non-tumor and tumor cores, consistent with its role in tumorigenesis. However, we did not find a clear inverse association between tumor ACK1 over-expression and survival, suggesting a more nuanced relationship to clinical outcomes. It is possible that majority of tumours on the TMA were stage I, where ACK1 expression may have less impact on clinical outcome. In later stage tumours, given the central role of ACK1 in migration and invasion, any increase in protein expression, regardless of extent, can be deleterious – thus mitigating any correlation between intensity percentage and patient outcomes. Intriguingly, over expression of ACK1 in paired surrounding adjacent “normal” lung tissue was associated with poor outcome, suggesting that ACK1 activation may be an early event in lung cancer carcinogenesis.

Another interesting observation arising in this study is the relationship between KRAS, ACK1 and cell migration, invasion and EMT. To date, there is no direct connection between the two proteins, with exception to the publication by Nur *et al.*[16]. Their work and ours suggested ACK1 is an important pro-oncogenic factor downstream of constitutive active RAS, probably also mediating EMT. Consistent with this notion, numerous reports have suggested that RAS activation by TGF signaling results in upregulation of 2 transcription factors, *snail* and *snug*, which repress E-cadherin and induce EMT [30-32]. Our preliminary analysis of *twist*, a mesenchymal transcription factor using real time PCR on our panel of NSCLC lines showed differential high and low *twist* mRNA in RAS mutant and WT cell lines respectively (data not shown). From this panel of cell lines, preliminary data suggests that RAS-driven EMT and migratory signals are mediated through ACK1.

Recently, the phase I study of bosutinib, including an expansion cohort in colon, lung and pancreatic cancer, was reported [20]. Although no objective tumor responses were recorded and the trial did not meet predetermined efficacy endpoints, stable disease was observed in 47% of NSCLC patients (n = 19), with 16% of patients with durable disease control of more than 24 weeks. Regrettably, KRAS and EGFR status were not reported in this study, and it is plausible that high ACK1 expression may stratify a subgroup of EGFR and KRAS mutant patients likely to respond to bosutinib.

In conclusion, ACK1 inhibition affects cell motility and migration particularly in the context of KRAS mutant NSCLC cell lines, and is highly expressed in lung adenocarcinoma as compared to paired surrounding non-tumor tissue. Given the implicated role of ACK-1 in EGFR, KRAS and ALK signaling, we suggest a potential therapeutic niche for combination studies with bosutinib selecting for patients with ACK-1 overexpression, specifically in circumventing tumor metastasis and recurrence.

## Materials and methods

### Lung cancer cell lines

Human lung adenocarcinoma cell lines [NCI-H1650, NCI-H1792, NCI-H1838, NCI-H1975, NCI-H2009, NCI-H2291, NCI-H2444, HCC-95, HCC-193, EKVX and SK-Lu-1] were provided by Max Planck Institute of Biochemistry and authenticated using STR profiling by John Hopkins Fragment Analysis Facility (April 2013). All cells were maintained in Roswell Park Memorial Institute medium supplemented with L-glutamine and 10% fetal bovine serum, except for SK-Lu-1 cells which were maintained in Minimum Essential Medium with L-glutamine and 10% fetal bovine serum. Normal Human Bronchial Epithelial Cells (NHBE) were purchased from Lonza (Basel, Switzerland), and cultured in serum-free medium as described by Clonetics. All cells were incubated in a 37°C incubator with 5% CO_2_.

### Reagents

Bosutinib (SKI-606) was purchased from LC Laboratories (Woburn, MA) and prepared in DMSO as 10 mM stock. The compound was diluted to desired concentration in culture medium (or DMSO) before treatment. Results were separately validated with bosutinib, synthesized by Vichem Chemie Research Ltd (Budapest, Hungary)

### Protein extraction and Western blot analysis

Cells were lysed in lysis buffer (50 mM HEPES, 150 mM NaCl, 1 mM EDTA, 10% Glycerin, 1% Triton X-100, 10 mM Sodium pyrophosphate, 10 mM sodium fluoride, 2 mM sodium orthovanadate, 1 mM phenylmethanesulfonyl fluoride, 0.2 μg/ml Aprotinin) for 20 minutes at 4°C, and protein concentration were measured using BCA protein assay (Thermo Scientific, Rockford, IL). Protein samples were subjected to SDS-PAGE and transferred onto polyvinylidene difluoride membrane. Membranes were blocked with 5% milk in PBST/TBST (0.1% Tween-20) and incubated with the primary antibodies: anti-ACK1 (A-11) antibodies (Santa Cruz Biotechnology, Santa Cruz, CA), anti-β-actin-HRP, anti-GAPDH-HRP, and anti-SRC antibodies (Cell Signaling Technology®, Beverly, MA). The secondary antibodies were anti-mouse and anti-rabbit horseradish peroxidase conjugated antibodies (GE Healthcare). Detection was performed using Immobilon Western Chemiluminescence HRP substrate (Millipore, Billerica, MA).

### Cell viability assay

Cancer cell lines and NHBE cells were seeded onto 96-well plates at 80% confluency (1000 to 5000 cells). Cells were treated with various concentrations of Bosutinib for 72 h. Cell viability was measured using CellTiter-Glo reagent (Promega, Madison, WI) according to manufacturer’s instructions. Cell viability results were normalized to DMSO-treated controls and expressed in percentage.

### Apoptotic assay

Cells were seeded onto 96-well plates at 80% confluency overnight (1000–5000 cells) and treated with Bosutinib at various concentrations for 72 h. Apoptosis was measured using Caspase-Glo® 3/7 luminescent assay according to manufacturer’s instructions (Promega Corp., Madison, WI). Caspase activity was expressed in percentage with DMSO treated cells set as 100%.

### Migration and invasion assay

Serum-starved cells (50000 to 100000) were suspended in 200 μL of serum-free medium and treated with Bosutinib (0.1, 0.5 and 1 μM) or DMSO control. Cells were loaded into each 8.0 μm pore polycarbonate membrane insert (Corning, Inc., Corning, NY), in duplicates. Cell migrated towards the lower chamber containing 600 μL of medium with 10% FBS and DMSO or Bosutinib (0.1, 0.5 and 1 μM) for 6 h. This is followed by fixation and staining with 0.5% crystal violet blue in 25% methanol. Non-migrating cells were removed with cotton swab and migrated cells were counted in five random microscopic fields. Invasion assay was performed as migration assay by replacing the transwell with Matrigel™ transwells (Corning, Inc., Corning, NY).

### siRNA silencing

Cells were seeded onto 24-well plate and transfected with siRNA against ACK1 (#103419), SRC (#683), or negative control (Ambion, Austin, TX) using Oligofectatmine™ transfection reagent (Invitrogen, Carlsbad, CA) as described in manufacturer’s protocol. Cells were collected for use at 72 h after transfection.

### Quantitative Real-Time PCR

Total RNA was extracted and purified using RNeasy Mini Kit (Qiagen, Germantown, MD). Superscript™ III First-strand synthesis kit ((Invitrogen, Carlsbad, CA) was used for reverse transcription under manufacturer’s recommended conditions. Real-time PCR from the cDNA product was performed with Applied Biosystems’s 7500 Fast real-time PCR system (Foster City, CA). Primers specific to *ACK1*, *SRC* and *GAPDH* were used together with Fast SYBR® green master mix (Applied Biosystems, Foster City, CA) for PCR amplification under manufacturer’s conditions.

### Zebrafish metastasis model

The zebrafish metastasis model (Zgraft) was developed with approved protocols from both the Wayne State University Institutional Animal Care and Use Committee (USA) and the University of Windsor Animal Care Committee, Canadian Council on Animal Care. Embryos were obtained from natural spawnings of an *AB* strain. Developmental stages are reported as hours post-fertilization (hpf) at 28°C.

NCI-H2009 tumor cells, transfected with siRNA against ACK1, SRC, or negative control, were each cultured at 37°C to 80% confluency, detached using Trypsin-EDTA solution (Sigma), washed twice with DPBS (Gibco, Invitrogen) and further incubated for 20 min at 37°C in serum free culture media containing 0.05% DiD (Vibrant, Invitrogen). After an incubation of 20 min, cells were washed twice and further incubated for 5 h in media containing 0.25 μM bosutinib. Cells were washed twice and re-suspended in DPBS for injection. 24–30 hpf zebrafish embryos were dechorionated and anesthetized with tricaine (Sigma). Using Nanoject II (Drummond) injector, 200 cells were injected into the yolk of each embryo. After injection, embryos were incubated for 2 h at 31°C and checked for successful injection. Embryos with fluorescent cells outside the yolk sack were excluded from further experimentation and analysis. Injected embryos were transferred to a 96-well plate (one embryo/well) containing Bosutinib (0.25 μM) diluted in 200 ml E3 media (without methylene blue) and further incubated at 35°C for 46 h. Control embryos without drug were treated and incubated in the diluent (DMSO).

### Quantification of cellular migration and metastasis in zebrafish

Cellular migration and metastasis of xenotransplanted tumor cells in zebrafish embryos is greatly influenced by the number of cells injected, cell survival during drug treatment, and the time of incubation post injection. For consistent quantitative comparison of cellular migration between different tumor cell lines and different drug treatments, a Migration Index is calculated from the analysis performed through microscopic imaging. Embryos are anesthetized on a microscope slide at 48 h post injection and drug treatment and imaged using confocal fluorescence microscope (Olympus, FV1200). Multiple Z plane images are taken of each embryo and composite images are made using ImageJ software. All composite images are gathered, aligned to a specific orientation, and analyzed as a scatter plot to determine tumor foci position relative to injection site (0,0 on graph). Number of metastatic tumor cell foci positions was calculated by applying a minimum area filter to exclude unanchored migrating single cells. Axes represent the distance (μm) from the injection site. Cumulative distance (CD) traveled by metastatic tumor cell foci (in μm) was therefore calculated from the graph as the sum of distances of all identified tumor foci from the injection point in a single embryo.

Migration Index = 1/n ∑(CD at 48 h/Total number of viable cells at 48 h) where n is the number of embryos considered in the experiment.

To quantify the surviving tumor cells in zebrafish larvae, zebrafish embryos were incubated in 50 μl protease solution (2 units/ml collagenase (Roche) and 60 units/ml dispase (Roche) in DMEM for 2 h at 37°C. Cells were gently dispersed with pipetting to dissociate transplanted embryo to a single cell suspension. 50 μl of 8% paraformaldehyde solution was added directly to the wells to fix and count the fluorescent cells.

### Clinical data

Cases of resected lung adenocarcinoma over a 10 years period were retrieved from the lung cancer database maintained in National Cancer Centre Singapore. Clinical outcome data including overall and relapse-free survival were collected from the database. This retrospective outcome data was approved by the Singhealth ethics review committee.

### Construction of tissue microarray (TMA)

Paraffin embedded formalin fixed tissue blocks with paired slides were retrieved and reviewed, and representative tumor areas were selected for TMA construction. Selected tumor samples were punched out from tissue blocks using the Beecher microarrayer. Briefly, for each sample one morphologically non-tumor area adjacent to the tumour and four morphologically representative tumor areas were defined based on haematoxylin and eosin (HE)-stained sections. From each of these areas, a tissue cylinder was punched out from the blocks and transferred into a recipient paraffin block. Each cylinder had diameter 2.0 mm.

### Immunohistochemistry

4 mm sections were cut from TMA blocks and fished onto coated slides (POLYSINE, Menzel-glaser) in a similar orientation to facilitate evaluation. Sections were stained with anti-ACK1 antibody using optimized protocols. Briefly, paraffin sections of formalin-fixed tissue were stained for ACK1 using anti-ACK (sc-323 Santa Cruz Biotechnology) with 1:100 dilution. Sections were pretreated in citrate buffer (pH 6.0) at 110°C for 15 minutes with a pressure cooker. The staining was done with Dako Autostainer plus (Dako Colorado, Inc., USA) with fully automated immunohistochemical staining and visualization by Dako REAL™ EnVision™ Detection System (K5007). A breast cancer section was used as positive control according to antibody data sheet.

Stained slides were scanned using ScanScope digital scanners (Aperio, Vista, CA). Scanned slides were then viewed and scored using the Aperio Image Scope to determine intensity of immunostaining. ACK1 immunopositivity was defined as presence of brown cytoplasmic staining. Staining intensity was scored as 0, 1+, 2+ and 3+ (no, weak, moderate and strong staining, respectively). Percentage of positively stained tumor cells was assessed as proportion of total number of tumor cells present in the section. Intensity percentage score (IP) were documented. Intensity percentage score was defined as product of the maximum immunostaining intensity and percentage of tumor cells stained. Staining were scored independently by two certified pathologists (Dr Lim KH and Dr Takano A).

### Statistical analysis

Tumor and non-tumor ACK1 immunohistochemistry measures were compared using paired t-tests. Overall and relapse-free survival were compared in the context of Cox proportional hazards models, both individually and after adjusting for the important covariates stage and grade. Analysis was performed on R statistical software. Data from preclinical experiments were analysed and presented using Graphpad Prism version 6.0c.

## Abbreviations

ACK1: Activated Cdc42 associated kinase 1; UBA: Ubiquitin association domain; EGFR: Epidermal growth factor receptor; RCC: Renal clear cell carcinoma; EMT: Epithelial-mesenchymal transition; TNF: Tumor necrosis factor; HSP60: Heat shock protein 60.

## Competing interests

All authors declare no conflict of interest.

## Authors' contributions

DSW provided and managed the clinical samples. BH performed the statistical analysis. JMT and SCT did all the cell-based experiments. IS and HPL set up the zebrafish metastasis assay and analysed the data. SSK and MMMT performed the histological sectioning and IHC staining and AT and HKL provided professional pathological assessment of the staining. EHT and WTL provided their clinical input and advises. The work was performed under the support of AU and EHT. The manuscript is drafted by DSW and BTC. All authors read and approved the final manuscript.

## Supplementary Material

Additional file 1: Figure S1 Bosutinib inhibit cell migration and invasion is ACK1 dependent and SRC independent. SK-Lu-1 were transfected with various siRNA using Oligofectamines and harvested at 72 h. (A) Quantitative real time PCR analysis was performed using 20 ng cDNA and respective primer set for ack1 and src normalized to gapdh. The serum-starved cells were trysinized and seeded in the upper chamber of the Transwells (8 μm pore, B) or MatrixgelTM(C), in the presence of DMSO or 0.5 μM of bosutinib. Medium containing 10% FBS and DMSO or bosutinib (0.5 μM) was used as chemoattractant in the lower chamber. Cells were fixed and stained with 0.5% crystal violet blue after 6 h (migration, B) and 24 h (invasion, C). Cells that migrated across the filter were counted. Experiments were carried out in duplicates with five random fields counted. Click here for file

Additional file 2: FigureS2 Real time PCR analysis of ACK1 or SRC. NCI-H2009 were transfected with siRNA (control, ACK1 or SRC) using Oligofectamine for 72 h. The cells were harvested at 72 h. Quantitative real-time PCR analysis was performed using 20 ng of cDNA and respective primers set for *ack1* and *src* and normalized to *gapdh.* Click here for file

Additional file 3: Table S1 Individual relationships between OS or RFS and clinical variables as well as ACK1 measures in tumor and paired non-tumor tissues. Click here for file

Additional file 4: Figure S3 Bosutinib inhibition of cell migration is AXL independent. (A) 50 μg lysate protein was analyzed for total AXL protein using Western blot. 500 μg of the individual lysate was immunoprecipitated with anti-AXL (AF154) and immunoblot with antiphosphotyrosine (4G10). The PVDF membrane were stripped and re-blotted with anti-AXL (C-20). (B) NCI-H1792 was transfected with siRNA using Oligofectamine for 72 h. The serum starved cells were trysinized and seeded in the upper chamber of the Transwell (8 μm pore), in the presence of DMSO or bosutinib at various concentrations. Medium containing 10% FBS and DMSO or bosutinib was used as chemoattractant in the lower chamber. Cells were fixed and stained with 0.5% crystal violet blue after 6 h. Cells that migrated across the filter were counted. Experiments were carried out in duplicates with five random fields counted. Knockdown was confirmed on western blot analysis with 50 μg of the total protein on the right. Click here for file
