# Supplementary material for: Bosutinib inhibits migration and invasion via ack1 in kras mutant non-small cell lung cancer
Source: Mol Cancer. 2014 Jan 24;13:13. doi: 10.1186/1476-4598-13-13 (PMC3930897; doi:10.1186/1476-4598-13-13)
Supplement: Additional file 1: Figure S1 — Bosutinib inhibit cell migration and invasion is ACK1 dependent and SRC independent. SK-Lu-1 were transfected with various siRNA using Oligofectamines and harvested at 72 h. (A) Quantitative real time PCR analysis was performed using 20 ng cDNA and respective primer set for ack1 and src normalized to gapdh. The serum-starved cells were trysinized and seeded in the upper chamber of the Transwells (8 μm pore, B) or MatrixgelTM(C), in the presence of DMSO or 0.5 μM of bosutinib. Medium containing 10% FBS and DMSO or bosutinib (0.5 μM) was used as chemoattractant in the lower chamber. Cells were fixed and stained with 0.5% crystal violet blue after 6 h (migration, B) and 24 h (invasion, C). Cells that migrated across the filter were counted. Experiments were carried out in duplicates with five random fields counted. [file 1476-4598-13-13-S1.pdf]

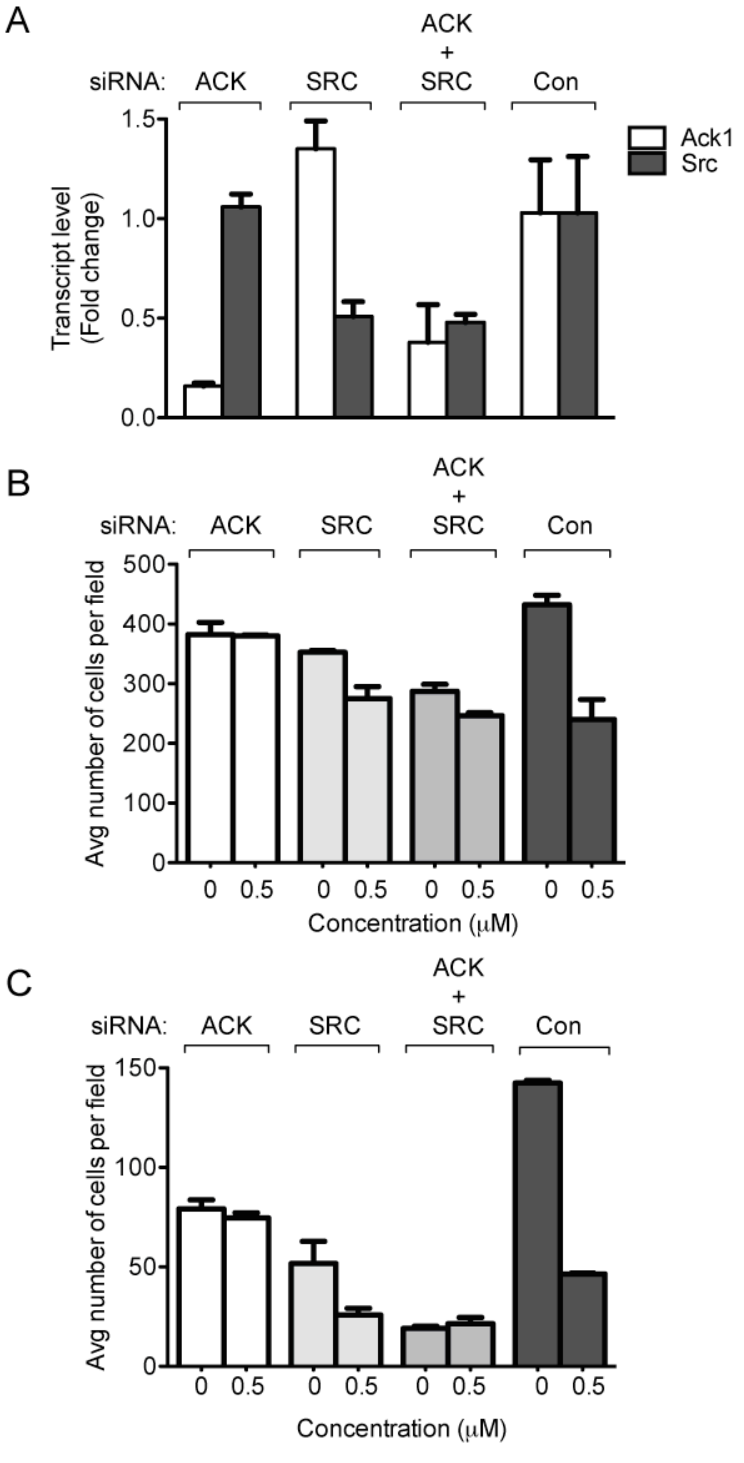

Supplementary fig S1. Bosutinib inhibit cell migration and invasion is ACK1 dependent and SRC independent. SK-Lu-1 were transfected with various siRNA using Oligofectamines and harvested at 72 h. (A) Quantitative real time PCR analysis was performed using 20 ng cDNA and respective primer set for ack1 and src normalized to gapdh. The serum-starved cells were trysinized and seeded in the upper chamber of the Transwells (8 μm pore, B) or Matrixgel^{TM}(C), in the presence of DMSO or 0.5 μM of bosutinib. Medium containing 10% FBS and DMSO or bosutinib (0.5 μM) was used as chemoattractant in the lower chamber. Cells were fixed and stained with 0.5% crystal violet blue after 6 h (migration, B) and 24 h (invasion, C). Cells that migrated across the filter were counted. Experiments were carried out in duplicates with five random fields counted.
