# Supplementary material for: Bosutinib inhibits migration and invasion via ack1 in kras mutant non-small cell lung cancer
Source: Mol Cancer. 2014 Jan 24;13:13. doi: 10.1186/1476-4598-13-13 (PMC3930897; doi:10.1186/1476-4598-13-13)
Supplement: Additional file 2: FigureS2 — Real time PCR analysis of ACK1 or SRC. NCI-H2009 were transfected with siRNA (control, ACK1 or SRC) using Oligofectamine for 72 h. The cells were harvested at 72 h. Quantitative real-time PCR analysis was performed using 20 ng of cDNA and respective primers set for ack1 and src and normalized to gapdh. [file 1476-4598-13-13-S2.pdf]

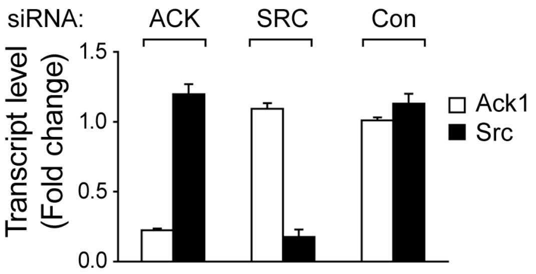

Supplementary fig S2. Real time PCR analysis of ACK1 or SRC. NCI-H2009 were transfected with siRNA (control, ACK1 or SRC) using Oligofectamine for 72 h. The cells were harvested at 72 h. Quantitative real-time PCR analysis was performed using 20 ng of cDNA and respective primers set for *ack1* and *src* and normalized to *gapdh.*
