# Supplementary material for: Bosutinib inhibits migration and invasion via ack1 in kras mutant non-small cell lung cancer
Source: Mol Cancer. 2014 Jan 24;13:13. doi: 10.1186/1476-4598-13-13 (PMC3930897; doi:10.1186/1476-4598-13-13)
Supplement: Additional file 3: Table S1 — Individual relationships between OS or RFS and clinical variables as well as ACK1 measures in tumor and paired non-tumor tissues. [file 1476-4598-13-13-S3.docx]

Table S1: Individual relationships between OS or RFS and clinical variables as well as ACK1 measures in tumor and paired non-tumor tissues.

^a^Number (percent) of those after stage 1. ^b^Number (percent) of those in stage 1. ^c^Number (percent) in each category out of non-missing; number (percent) missing out of total.

|  | **Overall Survival** | **Relapse-free Survival** |
| --- | --- | --- |
| **Stage**  Stage 1: 120 (57.1%)  Stage 2: 28 (13.3%)  Stage 3: 52 (24.8%)  Stage 4: 10 (4.8%) | p = 0.003 (overall)  Reference  HR: 1.344 (0.684, 2.642)  HR: 2.509 (1.521, 4.14)  HR: 2.893 (1.014, 8.258) | p = 0.004 (overall)  Reference  HR: 1.305 (0.664, 2.562)  HR: 2.417 (1.477, 3.956)  HR: 2.858 (1.001, 8.162) |
| **Grade**  Well: 35 (18.6%)  Moderate: 111 (59.0%)  Poor: 42 (22.3%)  Missing: 22 (10.5%) | p = 0.006 (overall)  Reference  HR: 2.174 (0.922, 5.126)  HR: 3.802 (1.529, 9.452) | p = 0.016 (overall)  Reference  HR: 1.957 (0.878, 4.362)  HR: 3.209 (1.358, 7.584) |
| **Adjuvant**  No: 173 (82.4%)  Yes: 37 (17.6%) | p = 0.320 (overall)  Reference  HR: 0.686 (0.314, 1.498) | p = 0.195 (overall)  Reference  HR: 0.617 (0.283, 1.346) |
| **Pathological T-Stage**^c^  T1: 71 (34.2%)  T2: 117 (56.5%)  T3/T4: 19 (9.2%)  Missing: 3 (1.4%) | p = 0.004 (overall)  Reference  HR: 1.509 (0.865, 2.631)  HR: 3.654 (1.769, 7.547) | p = 0.008 (overall)  Reference  HR: 1.477 (0.858, 2.543)  HR: 3.328 (1.626, 6.812) |
| **Pathological N-Stage**^c^  N0: 136 (65.4%)  N1: 25 (12.0%)  N2/N3: 47 (22.6%)  Missing: 2 (1.0%) | p = 0.006 (overall)  Reference  HR: 1.624 (0.837, 3.152)  HR: 2.39 (1.423, 4.013) | p = 0.005 (overall)  Reference  HR: 1.592 (0.821, 3.089)  HR: 2.384 (1.433, 3.967) |
| **Smoking Status**  Never Smoker: 119 (56.7%)  Ex-Smoker: 24 (11.4%)  Smoker: 67 (31.9%) | p = 0.014 (overall)  Reference  HR: 1.047 (0.555, 1.977)  HR: 2.075 (1.268, 3.396) | p = 0.047 (overall)  Reference  HR: 1.001 (0.532, 1.881)  HR: 1.827 (1.123, 2.972) |
| **Histology**  Papillary: 24 (11.4%)  Mucinous: 13 (6.2%)  Acinar: 129 (61.4%)  Solid: 44 (21.0%) | p = 0.032 (overall)  Reference  HR: 1.817 (0.452, 7.307)  HR: 2.852 (0.888, 9.159)  HR: 4.211 (1.253, 14.154) | p = 0.040 (overall)  Reference  HR: 2.262 (0.581, 8.802)  HR: 3.067 (0.956, 9.841)  HR: 4.295 (1.277, 14.442) |
| **Gender**  Female: 108 (51.4%)  Male: 102 (48.6%) | p = 0.178 (overall)  Reference  HR: 1.355 (0.87, 2.109) | p = 0.335 (overall)  Reference  HR: 1.24 (0.801, 1.918) |
| **Ethnicity**  Chinese: 181 (86.2%)  Non-Chinese: 29 (13.8%) | p = 0.393 (overall)  Reference  HR: 0.723 (0.332, 1.574) | p = 0.276 (overall)  Reference  HR: 0.665 (0.306, 1.446) |
| **Age**  Median: 62  Range: 27-84 | p = 0.777 (overall)  (per 10 years)  HR: 1.031 (0.836, 1.27) | p = 0.879 (overall)  (per 10 years)  HR: 1.016 (0.827, 1.249) |
| **ECOG**  Grade 0: 126 (60.0%)  Grading 1/2: 84 (40.0%) | p = 0.120 (overall)  Reference  HR: 1.424 (0.915, 2.216) | p = 0.109 (overall)  Reference  HR: 1.432 (0.925, 2.216) |
| **M-Stage**  M0: 200 (95.2%)  M1: 10 (4.8%) | p = 0.201 (overall)  Reference  HR: 2.088 (0.753, 5.789) | p = 0.204 (overall)  Reference  HR: 2.079 (0.749, 5.77) |
| **Tumor ACK1 IP**  Median: 90  Range: 20-210 | p = 0.659 (overall)  (per 10 units)  1.015 (0.951-1.084) | p = 0.883 (overall)  (per 10 units)  1.005 (0.94-1.075) |
| **Non-Tumor ACK1 IP**  Median: 20  Range: 0-40 | p = 0.037 (overall)  (per 10 units)  1.397 (1.022-1.908) | p = 0.030 (overall)  (per 10 units)  1.406 (1.035-1.911) |
