# Supplementary material for: Bosutinib inhibits migration and invasion via ack1 in kras mutant non-small cell lung cancer
Source: Mol Cancer. 2014 Jan 24;13:13. doi: 10.1186/1476-4598-13-13 (PMC3930897; doi:10.1186/1476-4598-13-13)
Supplement: Additional file 4: Figure S3 — Bosutinib inhibition of cell migration is AXL independent. (A) 50 μg lysate protein was analyzed for total AXL protein using Western blot. 500 μg of the individual lysate was immunoprecipitated with anti-AXL (AF154) and immunoblot with antiphosphotyrosine (4G10). The PVDF membrane were stripped and re-blotted with anti-AXL (C-20). (B) NCI-H1792 was transfected with siRNA using Oligofectamine for 72 h. The serum starved cells were trysinized and seeded in the upper chamber of the Transwell (8 μm pore), in the presence of DMSO or bosutinib at various concentrations. Medium containing 10% FBS and DMSO or bosutinib was used as chemoattractant in the lower chamber. Cells were fixed and stained with 0.5% crystal violet blue after 6 h. Cells that migrated across the filter were counted. Experiments were carried out in duplicates with five random fields counted. Knockdown was confirmed on western blot analysis with 50 μg of the total protein on the right. [file 1476-4598-13-13-S4.pdf]

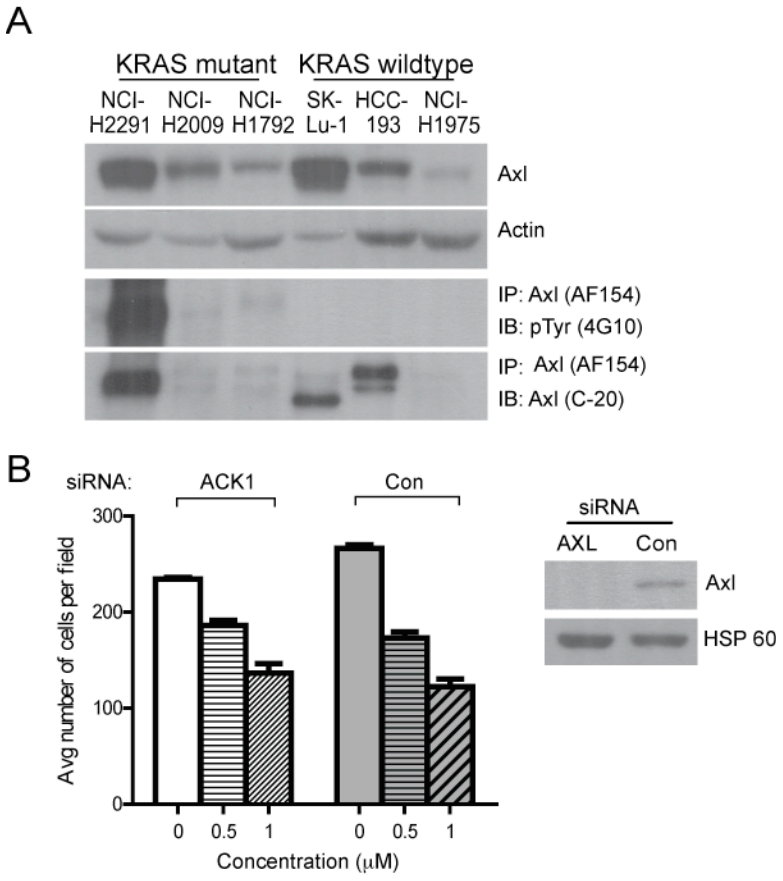

Supplementary fig S3. Bosutinib inhibition of cell migration is AXL independent. (A) 50 μg lysate protein was analyzed for total AXL protein using Western blot. 500 μg of the individual lysate was immunoprecipitated with anti-AXL (AF154) and immunoblot with anti-phosphotyrosine (4G10). The PVDF membrane were stripped and re-blotted with anti-AXL (C-20). (B) NCI-H1792 was transfected with siRNA using Oligofectamine for 72 h. The serum starved cells were trysinized and seeded in the upper chamber of the Transwell (8 μm pore), in the presence of DMSO or bosutinib at various concentrations. Medium containing 10% FBS and DMSO or bosutinib was used as chemoattractant in the lower chamber. Cells were fixed and stained with 0.5% crystal violet blue after 6 h. Cells that migrated across the filter were counted. Experiments were carried out in duplicates with five random fields counted. Knockdown was confirmed on western blot analysis with 50 μg of the total protein on the right .
